# Navigating virtual conferences as a junior researcher

**DOI:** 10.1038/s41467-020-18656-6

**Published:** 2020-10-07

## Abstract

Dr. Kishana Taylor (a microbiologist and Postdoctoral Researcher at the University of California, Davis), Dr. Nella Vargas-Barbosa (an electrochemist and Scientist at the Max Planck Institute) and Dr. Anouk Beniest (a geologist and Postdoctoral Researcher at GEOMAR Helmholtz Centre for Ocean Research Kiel) talked to *Nature Communications* about their recent experiences at virtual conferences as early career researchers, since the onset of international COVID-19 travel restrictions. Kishana, Nella and Anouk share tips for navigating virtual conferences as junior researchers, and they also give suggestions for conference organizers to improve virtual scientific meetings so they more inclusive for younger scientists.

**Figure:**
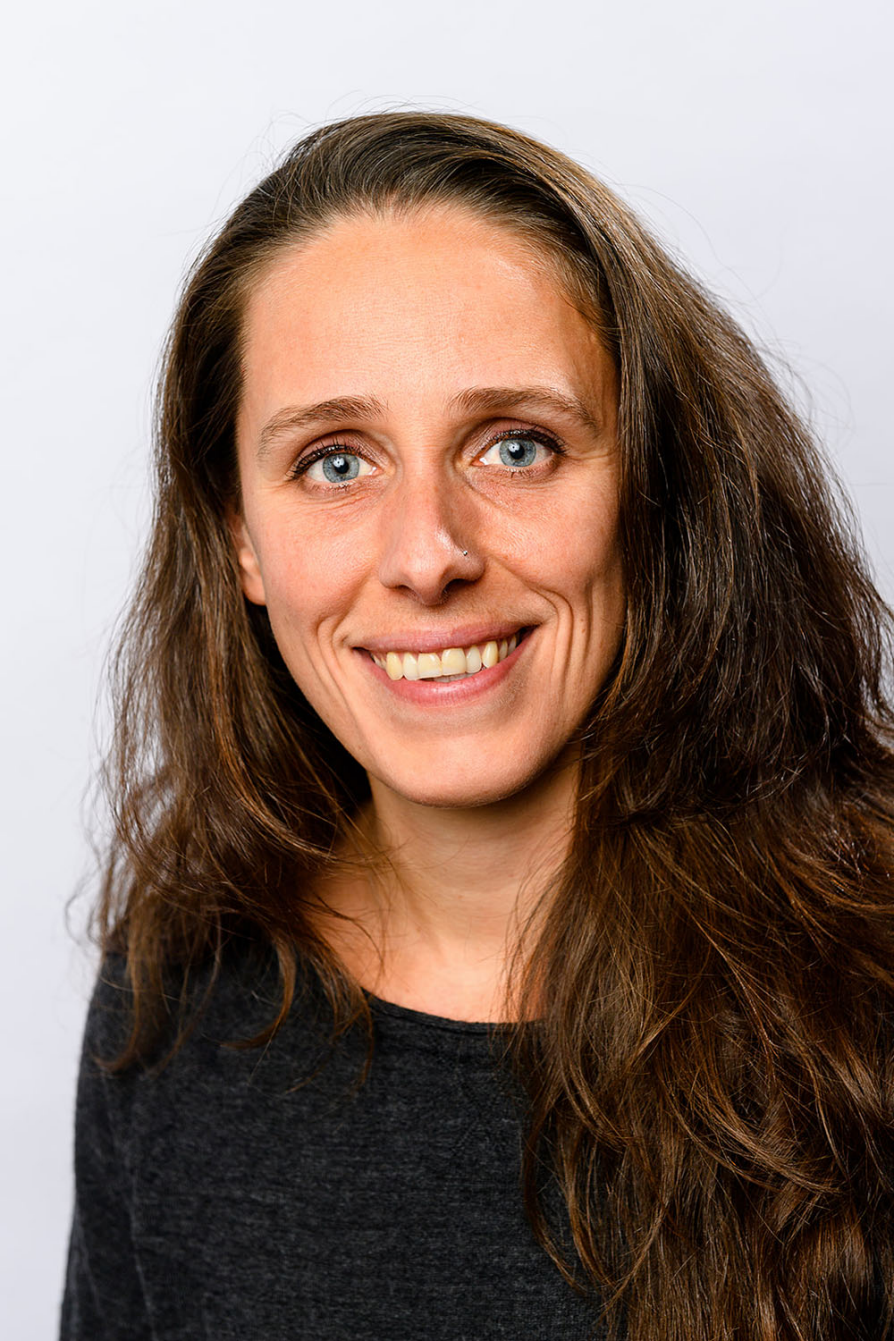
Dr. Anouk Beniest

**Figure:**
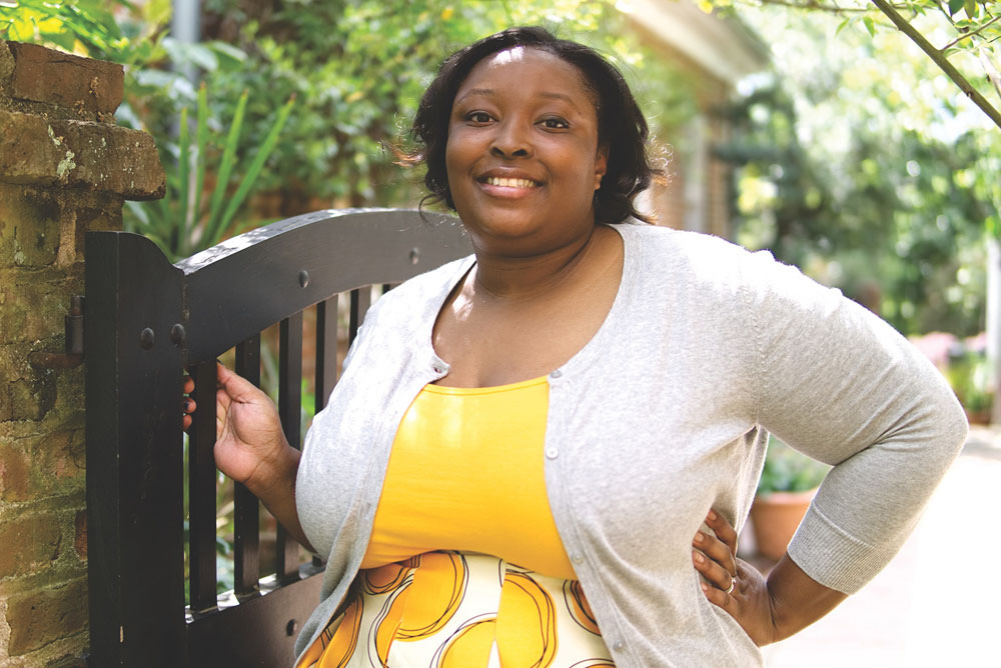
Dr. Kishana Taylor, Doanise Thompson

**Figure:**
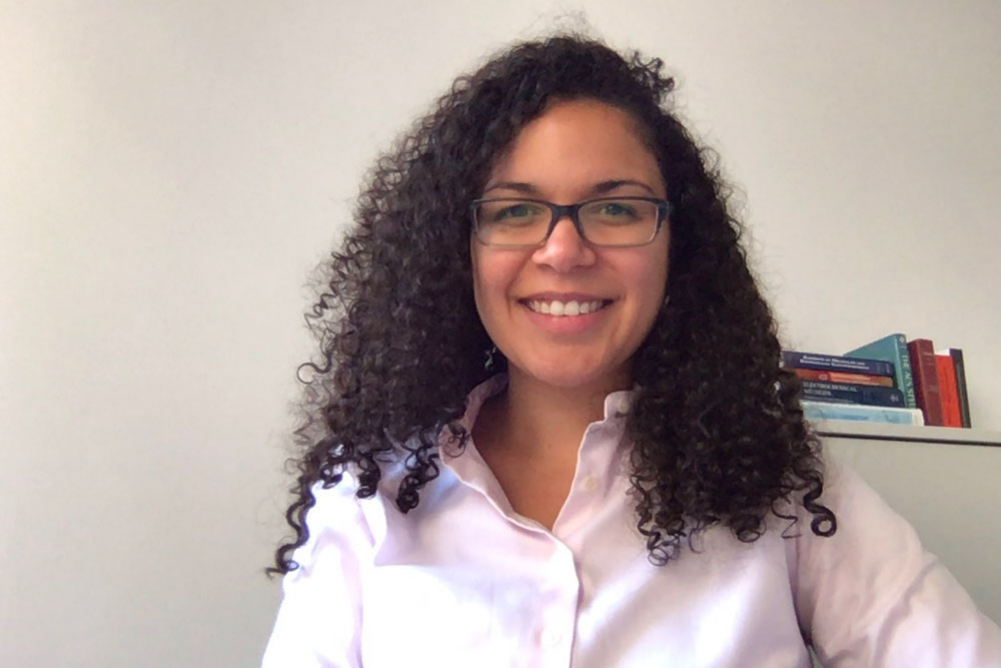
Dr. Nella Vargas-Barbosa

Please tell us about some of your recent experiences as an Early Career Researcher attendee at a virtual conference, and give us a bit of background on any research you presented.

**Nella**: My work from home period started in early March, and since then I have presented our group’s research in a CellPress Webinar on The Future of Batteries (hosted by Joule and Cell Reports Physical Sciences), and participated as an attendee at two other virtual conferences related to Next Generation Electrochemistry. Although our general research interests are on interfacial (photo)electrochemistry, almost all of these events were focused on our recent work on solid electrolytes for all solid-state batteries.

**Kishana**: I have attended several virtual conferences in the past few months, American Society for Virology (ASV), American Society for Microbiology (ASM), Microbe and ASM Conference for Undergraduate Education (ASMCUE). ASV hosted its planned workshops virtually, while ASM spread out the content over a number of weeks, but had plenary and keynote speakers live in addition to symposia (both live and video recorded). ASMCUE had virtual poster presentations with built in networking sessions in addition to keynotes and symposia. I personally did not present any research this year, but I have visited virtual posters and also watched plenary sessions and talks.

**Anouk**: My first virtual conference experience was Sharing Geoscience Online, the virtual General Assembly of the European Geosciences Union (EGU). The conference used a text-based chat as a communication tool to discuss the presentations, which were uploaded a few weeks before the meeting. During the session each presenter had a few minutes to introduce their work in the text-chat and answer questions. For sharing my science, this digital format was fantastic, however, the lack of any form of visual interaction with peers and others, made it more difficult to network. I prepared a presentation on the first offshore geological map of the Scotia Sea area. It was interactive so that people could jump straight to the aspect of the presentation they were interested in. This new way of sharing science required a bit more preparation, as I had to look at other virtual presentation styles beforehand and to prepare some questions in advance for the chat session. But, because the whole conference was online, it was also easy to look for additional background information or download a paper of the presented work during the meeting.

Do you have any tips for preparing a virtual presentation? What do you think your colleagues can do to help make their presentation stand out to potential future employers or collaborators?

**Kishana**: I felt that the best presentations engaged with the audience more than your typical talk. While being able to engage with your audience can depend heavily on what type of research you are presenting, things like conducting polls with the audience went well and can increase the focus on your presentation. This can especially integrate well with virtual posters, which in essence are now mini-talks. I would also suggest getting to know the virtual platform that you will be presenting from before the meeting starts. This advice can go for both presenters and general attendees. There were a few times when I was behind the curve and didn’t know how to respond to a poll, or raise my hand (virtually) to ask a question to a presenter etc., which threw my focus off and it made it hard to pay attention for a few moments as I was scrambling to figure out the technicalities.

**Anouk**: To really stand out as an early career researcher, try to be as creative as possible and think beyond the conventional presentation styles, include visuals, audio-recordings and hyperlinks (for example, that lead to your personal website, the papers you are citing, explanation movies, etc.). Once the virtual conference is finished, make sure your presentation remains visible online. In some cases, the conference organization has the possibility to archive your presentation online, if not, you can always consider self-archiving your presentation.

For talks, which had to be uploaded in advance, some scientists presented their research in the form of a short movie, which I think is a very clever and efficient way to present your work, as it allows you to include visuals, written text and audio explanations. Since everyone learns differently you will be able to reach many people through their preferred way of receiving information. Posters can be tricky, as generally the screen that readers will access the presentation from are too small to show the whole poster, which can make the poster very difficult to read. Presenters should ensure the audiences’ average screen size is taken into account when making a poster presentation.

**Nella**: Tip #1: Less (per slide) is more. Keeping the number of plots/schemes at ~2 or 3 maximum will allow the audience to “see” what you’re talking about. Some of the available virtual platforms are not designed for presentations of scientific results, so filling up the slides can make it difficult for your audience to read the data themselves. Not everybody has a high-res screen available, therefore, make the axis titles and labels larger than usual, the same for data points.

Tip #2: Each slide has a single-sentence take-home message. Many of us are juggling various tasks (e.g. childcare, important emails/calls, necessary house repairs) while “listening in” on a talk. To make sure that your audience can catch-up after a few seconds of distraction, the take home message is somewhere (preferably always in the same location) in each slide.

Tip #3: Do your homework about the expected audience. If it is an experienced collaborator in your field, you do not need to spend 1/3^rd^ of the talk on a general introduction about why the research you are presenting is important. Jump right in on the work you have done so far and open yourself to the discussion. If you are presenting to a more interdisciplinary audience, make the slides more didactic and build-up your topic. For these audiences, a qualitative description of the experimental approaches and results is usually sufficient. Any experts in the audience can then ask more details during the Q&A session.

In terms of networking, how can early career researchers coordinate virtual introductions or e-meet potential future collaborators at online meetings?

**Anouk**: Young researchers rely on their network not only to start collaborations and to find new positions, but also to exchange information about career opportunities, how to maintain a work-life balance, and tips and tricks for moving to a different country. Your network gives you a sense of security as well, but if your network is not maintained it may lose its power. Expanding your network in a virtual environment requires more pro-activeness from your side. There is less chance you may ‘run’ into someone, so taking initiative is key. You need to be prepared with a virtual business card. This can be anything from your personal institute webpage, or a profile on ResearchGate or Google Scholar, or an active Twitter or Instagram account, or your personal website.

I also believe there is a responsibility for senior scientists to join virtual networking events directed at early career researchers. In a virtual environment it takes more effort to invest in the next generation, but maintaining this network across all career stages will pay off as future scientists will be more informed, the academic landscape will be more inclusive, and the science more rigorous. But there are of course more people you may want to include in your network, for example, people from the industry, journalists or politicians. To make networking online as efficient and fruitful as networking at a physical conference, all parties need to be actively involved.

**Nella**: I would suggest to early career researchers to be bold and send out an email or direct message (DM) via Twitter to whomever they want to network/work with. This is how I got my Ph.D., post-doc and junior group lead positions. Tell them you are interested in learning more about their work and if they are available/willing to let you present some of your work at group meeting. Especially at this time, during many pandemic-induced lab shutdowns, most groups are running out of new results generated internally to discuss, so an invited speaker can be quite refreshing.

**Kishana**: Some conferences will have virtual networking and breakout sessions based on topic of interest. If you are attending a virtual conference that does this, these are perfect opportunities to network with other attendees. It’s similar to traditional conference networking in that most online platforms (Zoom, Teams, etc.) will have a name displayed in place of name tags. However, the virtual environment can be a bit more advantageous because it’s easier to spot the people you may be most interested in speaking with. Once you have identified people you wish to e-meet, there are the private and public chat features (e.g. on Zoom) where you can send these people a message. I would suggest you be just as formal/informal as you would be in person and remember that the conference organizers will receive a transcript of whatever conversations you have on that platform.

Often the more casual poster sessions and evening social activities are where most introductions and connections are made at scientific meetings, from your experience, how were the more informal aspects of conferences replicated in virtual form? Were there any particular events for networking that worked well for you?

**Kishana**: Not all conferences will simulate the social activities. So, if your conference does offer this option, take advantage! For the virtual conferences that did simulate these activities, things like happy hours, breaks and networking sessions were simulated via breakout rooms or separately scheduled calls. In all of these situations, participants were asked to introduce themselves either in the chat box or on video. This was great because in a traditional meeting this doesn’t always happen. You can use this to your advantage because it will be easier to pinpoint people who have similar interests to you. I was able to message with some conference attendees that I both know in real life and also people I have interacted with previously online through social media accounts, like Twitter. I found that the scheduled separate call times for networking sessions and fire side chats were the most effective for early career researchers. Typically, there were less participants than on the actual presentations and more time to make personal introductions.

**Anouk**: The format of the networking event can make or break it. I often feel there is not enough time to exchange the necessary information to get acquainted with people. Some events make it hard to leave a virtual space, which then prevents you from checking out another virtual spaces and chat with people there. Other events require you to plan well ahead without allowing last-minute change. This really takes away the spontaneity of participating in an event in general. At the ‘Sharing Geoscience Online’ the various subject divisions organized separate networking events directed at younger researchers. During the event we were divided randomly into smaller groups to allow us to chat with new people. I thought that simulated the more casual parts of a physical conference nicely.

If you find yourself in a larger group during a networking event, I really recommend to use a text-based chatting tool to communicate, as you can quickly comment on remarks made by the speaker. This can result in very nice and lively discussions and it also substitutes to some extent the small talk at the posters or the hallway of a conference center. All this chat-based communication does require you to be fast with your fingers, so upping your skills with a typing course is a must.

**Nella**: I have yet to participate in a virtual poster session event. I have seen them promoted via hashtags on twitter but seem to be very time-consuming because you have to “be there” at a certain time (when the presenter is also there), which may be inconvenient due to time differences. I am also not sure the character count limit of Twitter can lead to productive scientific discussions. Moreover, events held on certain social media platforms are not fully accessible to scientists that are physically located in countries with special internet regulations and, therefore, online scientific events and discussions held exclusively on such platforms are not a sustainable approach to ensure full international inclusivity.

In the case of the informal introductions during webinars, I found it helpful that we (the speakers) were asked to meet with the organizers 30 min before the event started. During this time, we tested audio capabilities, we discussed how the event would run, tested the slides, and then the speakers got to just chat and catch up until the event started. The event ran super smoothly. I already knew the other two speakers, but if I hadn’t I think it would’ve been a great opportunity to build my network.

Finally, I have yet to participate in a #SocialIsolationSocial organized by Stuart Cantrill (@stuartcantrill) and Louis-Charles Campeau (@DrLCsquare), but they seem to really bring people together. Similar to my suggestion above, just message people and schedule an opportunity to meet.

Do you have any suggestions for conference organizers to improve virtual meetings so they are friendlier to younger scientists? For example, are there any particular virtual meeting formats you think are better or worse than other formats?

**Nella**: I would say that there needs to be a better way to handle the discussions at virtual meetings. Sometimes people can ask questions directly, sometimes only via the chat function, sometimes not at all. This is of course very challenging because the number of participants is often very large. But maybe there needs to be a post-talk session in a break out room where speakers can chat with the members of the audience who are interested in further details.

To make it more friendly to early career researchers, well, invite them to give talks. Yes, presentations from the established people in the field are great for increasing clicks and audience members, but it is often the group leads, post-docs and graduate students that really know the details of the projects. Organizers can email the established researchers and ask them to suggest their trainees to present on the specific topic. They will appreciate the opportunity, and the established researchers will appreciate not having to schedule (yet another) virtual conference.

**Kishana**: My suggestions for conference organizers would be to make sure that you are making time for events for early career researchers. Schedule that separate networking session or let participants know that you encourage them to reach out on the platforms, and to please respond and encourage this type of activity while attending the conference. For first time attendees, orientation events would be a great way to let everyone know what the schedule of the meeting is or what the expectations are in terms of virtual networking etiquette. I know that I am more likely to be an active participant when I know the proper protocols and etiquette.

**Anouk**: For the scientific presentations and discussions, a fluid virtual platform is needed where it is easy to follow and participate in the sessions, and where topical discussions can be held in bigger groups that allow everyone to enter the discussion and share their opinion, i.e. by using a text-based chatting tool. Most of all it should work properly. Technical failure is not an option, especially not when participants have to pay a conference fee. I am in favor of hosting these events for free as it allows scientists from all over the world to participate. Virtual meetings and conferences are arguably one of the most inclusive spaces on Earth, provided that participation fee is kept to a minimum and the chosen platform allows for slower internet connections.

For networking events or workshops, a format that I think works well is where there is a main virtual environment where people can always return to. From there, the moderators can either appoint or divide people into smaller groups, or open virtual spaces, perhaps with specific topics, where people can choose to go to or not. Organizers and moderators should encourage participants to turn on their cameras during video meetings, or, when the internet connection is not sufficiently stable for that, ask them to have a profile picture of themselves so that everyone can put faces to names. Smaller groups of 4–5 people can work fairly well and allows more informal and inclusive conversations as all participants get a chance to speak. When the groups get bigger, for example during a round-table discussion, it is really essential to have a good moderator who has a schedule, watches the time and makes sure everyone has a chance to speak. Another format that I found very efficient and fun are ‘speed-dating’-type events, with short 5–6 min 1-to-1 conversations. In this case you both need to talk so it is easier to get to know each other and to share your virtual business card.

